# Psychogenic non-epileptic seizures in youth: Individual and family psychiatric characteristics

**DOI:** 10.3389/fpsyt.2022.1068439

**Published:** 2022-12-15

**Authors:** Julia Doss

**Affiliations:** ^1^Julia Doss Clinic of Health Psychology, LLC, Minneapolis, MN, United States; ^2^Children's Hospitals and Clinics of Minnesota, Saint Paul, MN, United States

**Keywords:** psychogenic non-epileptic seizures, functional seizures, conversion disorder, pseudoseizures, Functional Neurological Disorder

## Abstract

**Introduction:**

Youth with psychogenic non-epileptic seizures (PNES) are an understudied group associated with significant medical and psychiatric morbidity. Several studies have examined characteristics associated with youth's development of this disorder, though the exploration of family factors including psychiatric illness, has been lacking. This study sought to establish the need for a more comprehensive future study.

**Methods:**

A retrospective chart review was conducted on patients who had been admitted and diagnosed with PNES at the epilepsy monitoring unit at Children's Hospitals and Clinics of Minnesota. A total of 62 patients were included. All patients were evaluated by an epileptologist and psychologist during their diagnostic admission. “Spells” in question were captured *via* video EEG monitoring. PNES youth and family risk factors were assessed.

**Results:**

Mean age of PNES symptom onset was 13.9 years. Patients (73%) were diagnosed within 6 months of onset of symptoms. Histories of other impairing somatic complaints were present in the youth (54%), with 67% having prior psychiatric diagnoses. Experiencing suicidal ideation or thoughts of self-harm occurred in 47% of this sample. Family members were unaware of the history of these symptoms with 12% of the parent's reporting awareness. Family history of psychiatric disorders (first-degree relatives of patient) was present in 54% of the sample, with anxiety, depression and conversion disorder being the most commonly endorsed diagnoses.

**Conclusions:**

Youth with PNES present with comorbid psychiatric disorders, though prior assessment and treatment for these disorders was not common. Youth with PNES have history of suicidal ideation and thoughts of self-harm, though parental awareness of these co-occurring symptoms is limited. Family risk factors, such as history of psychiatric disorder in first degree relatives, was high. The impact of these family risk factors is understudied and should be further evaluated to better understand the impact on development and maintenance of this disorder in youth.

## Introduction

Psychogenic non-epileptic seizures (PNES) are a complex biopsychosocial condition involving alteration in behavior, mood, perception and sensation that resemble epileptic seizures but are not due to epileptiform activity in the brain (1). Psychogenic seizures are classified as a Conversion Disorder under Functional Neurological Disorders (FND) in the Diagnostic and Statistical Manual of Mental Disorders, Fifth Edition (2) with video electroencephalogram the “gold standard” for diagnosis according to the International League Against Epilepsy task force (3). It is a heterogeneous disorder, with varied etiology and course. Youth with PNES are an understudied part of this group. The prevalence in youth is thought to be lower than in adults, though much of this data is estimated from admissions to comprehensive epilepsy centers and thought to be an underestimate of the overall population (4). The associated medical and psychological cost of the disorder and poor prognosis for future functioning if not treated, highlight the need for greater focus on this disorder in youth (5). Several studies have examined characteristics associated with youth's development of this disorder, though the exploration of family factors, including psychiatric illness, has been lacking (5–8).

It has been well established that youth with PNES experience co-occurring psychiatric disorders with anxiety, social anxiety in particular, and depression being the primary diagnoses (9, 10). These youth have not had prior mental health treatment, nor were they necessarily diagnosed with a psychiatric condition prior to the onset of PNES, though they experienced symptoms (5). It is not well understood why these youth are not diagnosed or treated sooner, but factors related to their Conversion Disorder could be at play, including a lack of insight or understanding of their emotional experiences (5).

Drastic changes to functioning are common following the onset of PNES in youth, commonly with increased medical utilization and decreased school attendance (5, 7). These costly disruptions often impact the family as well, resulting in significant challenges until the symptoms improve. Thus, a focus on symptom management in treatment is often a first priority, with returning to more independent functioning being a primary goal (11). Treatment studies of PNES have focused primarily on managing the PNES symptoms, and have not extensively examined the impact of the known risk factors for the disorder, namely underlying psychiatric conditions or management of family stressors (12).

Family functioning and family reaction to symptoms has an influence on the maintenance and management of PNES symptoms (12). Family coping strategies aimed at managing PNES have been shown to quickly improve functioning (12). While improvement in symptoms is the first goal, it is often not the only thing important in treatment (11). A recent study found that youth reported their PNES symptoms improved over time and they could return to more typical functioning, however improvement in symptoms of co-occurring anxiety or depression remained poor. In this same study, though the youth continued to report experiencing significant emotional struggles, parents of the youth reported that both functional and emotional improvement coincided (13). Parent's ability to accurately assess their child's emotional functioning is an important factor in management of mental health concerns (13).

Family history, family stressors, genetics and learned behavior likely play a role in this disorder as with other psychological conditions (14). In adults with FND, the literature has shown that certain “life factors” appeared to be significant in the development of the disorder. The “life factors” can include relationship struggles, death or bereavement, and medical or mental health factors (15). Family medical history, specifically history of epilepsy in a family members, has long been recognized as a potential model for learned behavior in the development of PNES, though recent studies have not found this to be a significant factor (16, 17).

Parental reaction to illness may have a strong influence in the development and maintenance of PNES symptoms. Based on family communication and learned behavior theories, conversion/functional symptoms in youth can develop in families with maladaptive response to illness, or if the youth is more supported or reinforced when they experience physical symptoms or behaviors, but do not receive the same attention/concern when presenting with emotional symptoms (18). Several studies have noted that parents of children with somatization or conversion symptoms may perceive their child as more medically vulnerable, and therefore, may be more likely to react when physical symptoms present (9, 18).

Understanding family experiences of PNES sheds some light on how family functioning may contribute to the diagnosis itself as well as the management of it. The youth's response to their symptoms, which often involves fear initially, can produce significant parental reactions. There may also be experiences of guilt on the youth's part for disruption that the symptoms cause in the family, while parents may feel guilty about not being able to have more influence over the symptoms (19). The financial burden, lost time in school, and disruption to daily routine cause significant impact and can result in greater emotional burden for both the youth and family (5, 19). When the family then experiences what they feel is dismissal of the serious nature of their symptoms by medical providers, defensiveness regarding how to proceed can form and result in treatment challenges and delays (20).

History of parental psychopathology is a well-known risk factor for the development of mental health disorders in youth, including functional or conversion disorders (21–23). Mothers of youth with PNES, as compared to youth with epilepsy, were found to have significantly higher depression and higher anxiety scores (9). In the few studies that exist examining mental health in youth with other somatic conditions, parents endorsed more symptoms of anxiety, depression and somatization than in parents of normal controls (21, 24). In addition, higher rates of psychopathology in family members has been reported in adults with PNES (25). Poor family communication and high conflict have also been reported in adults who developed PNES (25). Parental physical and emotional health, as well as that of siblings in the home, likely has significant impact on youth with PNES, though a comprehensive examination of these family risk factors has yet to be undertaken.

The aim of the current study was to examine physical and emotional health characteristics of a sample of youth with PNES. Personal risk factors of the youth with PNES, parental awareness of those emotional struggles and family risk factors, primarily presence of psychiatric disorders in first degree relatives, will be examined.

## Methods

### Participants

This retrospective chart review included 62 youth, aged 5–18, with a confirmed video EEG (vEEG) diagnosis of PNES and who were evaluated by a pediatric epileptologist. Participants were patients who had been admitted to the pediatric epilepsy unit at the Children's Hospitals and Clinics of Minnesota from 2008-2019. All patients underwent video EEG monitoring with recording of a “typical episode”. If necessary to rule out other medical conditions, evaluations (cardiac, other neurological) were completed prior to diagnosing PNES. Once PNES was established, all of the patients in this sample were then treated for the PNES by a psychologist at the Minnesota Epilepsy Group. Patients were excluded from this study if they had known cognitive impairment (IQ < 70), other types of non-epileptic events, and if they had non-English speaking parents. All patients were evaluated through semi-structured clinical interview by a pediatric psychologist at the time of the evaluation on the epilepsy monitoring unit.

**Figure 1:**
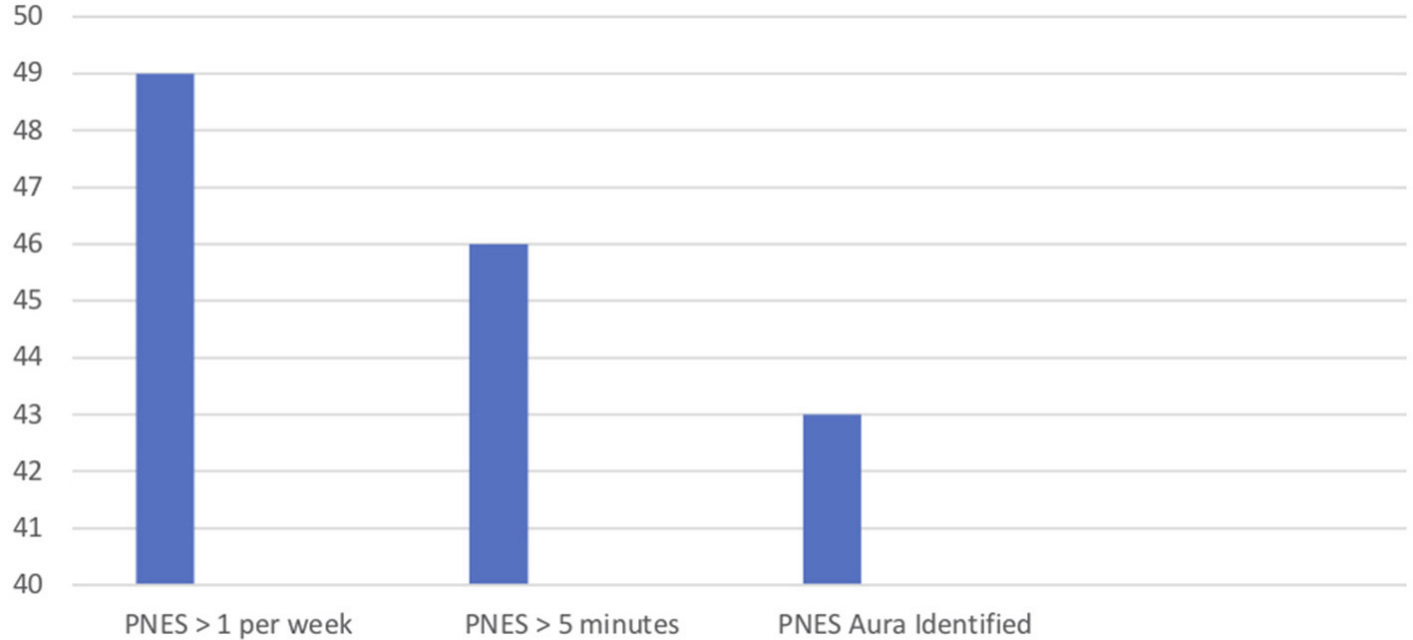
PNES characteristics at evaluation. 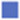
Number of patients.

**Figure 2:**
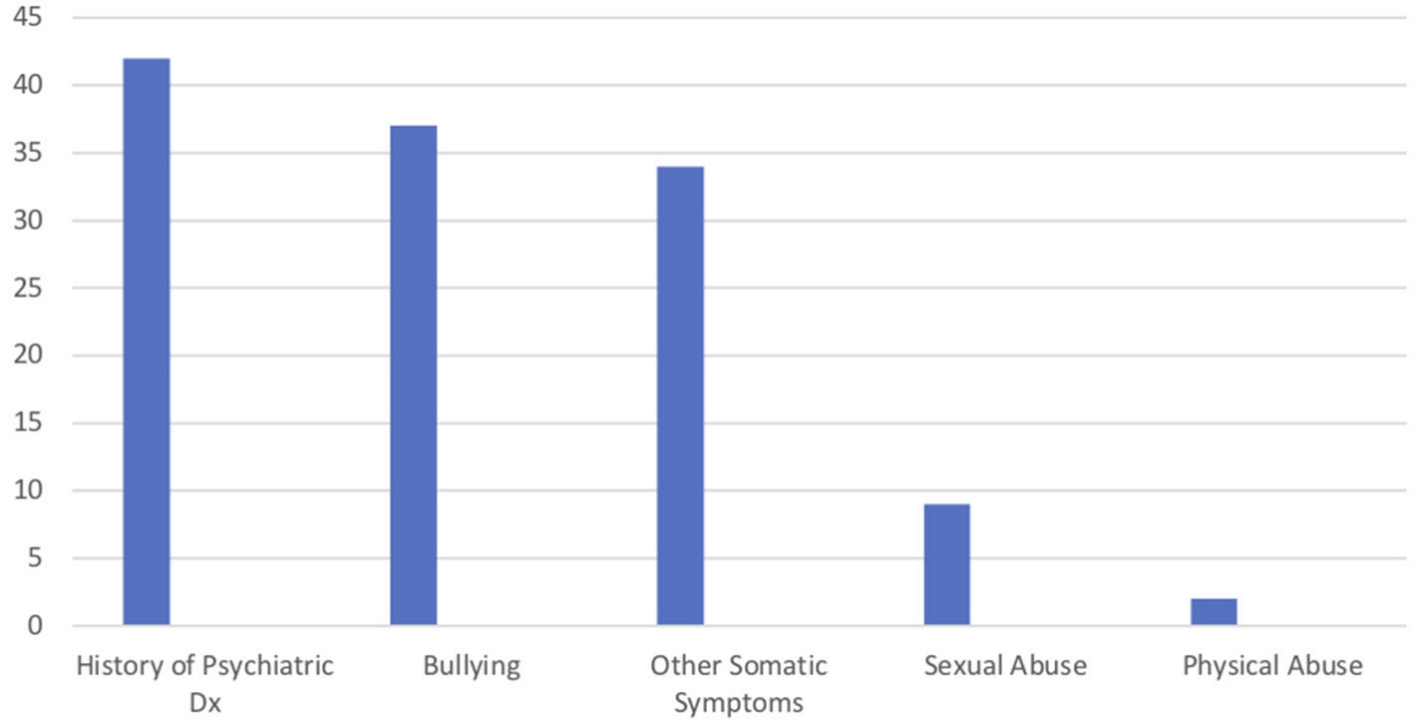
PNES patient risk factors. 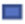 Number of patient's with risk factors.

**Figure 3:**
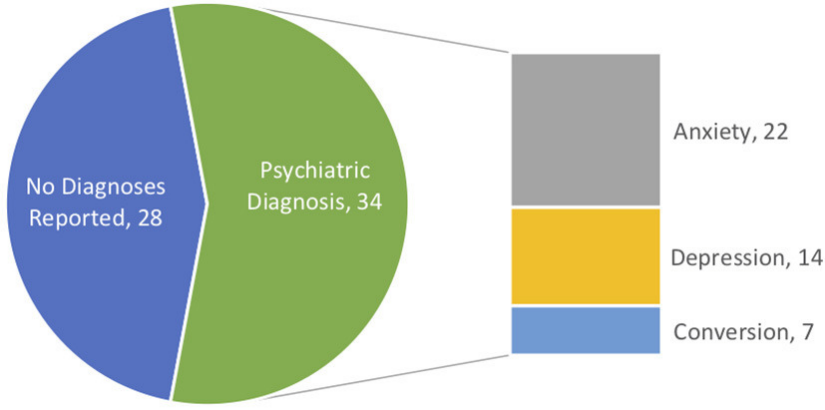
Family psychiatric history. 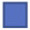 Family with no diagnosis. 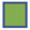 Family with diagnosis.

Data collected and evaluated for this study included: age, gender, medical history and prior psychological history and history of epilepsy (Table 1). Factors such as length, frequency and severity of the PNES itself were gathered (Figure 1). Youth PNES risk factors were assessed (Figure 2). In addition, family history, parental and sibling mental health and medical history were collected (Figure 3).

**Table 1:** Patient demographics.

Total sample	62
Age at PNES onset	13.9 y
Age range	5–18 y
Gender	48 females
Range from PNES onset to diagnosis	1 day−3 years
Diagnosed within 6 months of onset	76%
History of other impairing somatic complaints	55% (34/62)
History of Epilepsy	24% (15/62)
History of prior psychiatric diagnoses	68% (42/62)

## Results

Mean age of PNES symptom onset was 13.9 years, with 77% being female. Patients in this sample 73% (*n* = 45) were diagnosed within 6 months of onset of symptoms, with a mean of 26.97 weeks and a median of 8 weeks (1–156). There were 15 patients (24%) who had a history of epileptic seizures. Those with a delay of over 6 months between PNES onset and diagnostic evaluation were much more likely to have had prior extensive evaluations for their PNES symptoms (X^2^ = 9.94, *p* = 0.002).

At time of evaluation, PNES symptoms were frequent with 79% (*n* = 48) of the sample experiencing more than one PNES per week. The youth, 74% (*n* = 45), also experienced episodes longer than 5 minutes. An “aura,” or a set of symptoms that indicated the onset of the PNES, occurred for most of the patients as well (69%, *n* = 42) (Figure 1).

The PNES youth experienced a number of risk factors that may have contributed to the development of the diagnosis (Figure 2). At time of diagnosis, all patients met criteria for Functional Neurological Disorder and had co-occurring either anxiety or depression diagnoses. History of psychiatric diagnoses, present before the onset of PNES symptom, were found in 41 of the patients (66%), though only 19 (31%) had prior psychiatric treatment for their mental health symptoms. At time of evaluation, 29 PNES patients (47%) had experienced suicidal ideation or thoughts of self-harm, though parental awareness of the symptoms at the time of occurrence was low, with only eight parents reporting awareness of these symptoms. Patients (60%, *n* = 37), had experienced bullying in the past. While the medical evaluation on the monitoring unit was to evaluate the seizure-like symptoms, 54% (*n* = 33) of the patients reported histories of experiencing other impairing somatic complaints. Histories of sexual (20%, *n* = 9) and physical abuse (3%, *n* = 2) were also present.

Family history of psychiatric disorders (first-degree relatives of patient) was present, with 56% (*n* = 34) experiencing a psychiatric diagnosis during their lifetime. Of those, anxiety (22), depression (14), and Conversion disorder (7) were the most commonly reported diagnoses. Of those that did not endorse history of psychiatric illness, 21 reported that it was not present, while seven did not answer the diagnostic questions pertaining to family psychiatric disorder (Figure 3).

## Discussion

Youth with PNES are a heterogeneous group with a variety of risk factors thought to contribute to the development of the disorder (5). While there are several studies which have highlighted the prevalence of both anxiety and depressive disorders in youth with PNES, (5, 6) no known studies have examined the incidence of psychiatric disorders in first degree relatives of these youth, though it is well established that both genetics and learned behavior are significant risk factors in other mental health conditions (14, 20).

Similar to prior reports of co-occurring psychiatric disorders in youth with PNES (6), this study also found that 100% of the youth met criteria for a co-occurring psychiatric condition in addition to meeting criteria for Functional Neurological Disorder at the time of their PNES diagnosis. A significant number of the youth also had been diagnosed with a psychiatric condition prior to the onset of their PNES, but few had received treatment, a factor that has also been found in other studies (6). Untreated psychiatric illness, and the other personal risk factors, such as bullying, contribute to the development of PNES and can influence it's treatment (7). There were also low rates of physical and sexual abuse, which remains a risk factor, though has been shown to be prevalent than other risk factors in the pediatric population (5).

Another key finding of this study was that parental awareness of their child's other psychiatric symptoms was poor. While this has been reported in a prior studies (6, 7, 18), it continues to be an important factor in understanding the development and management of this disorder. It is not well understood why there is lack of awareness. Within the epilepsy population, family member's expressed emotion has been shown to influence course of illness and adjustment to seizures (26). In PNES, it may be due to the youth under-reporting or recognizing emotional symptoms, youth's difficulty expressing their emotional struggles, or caregivers' difficulty recognizing that their child is struggling. Future studies may further assess family communication and parental modeling of emotion and how this influences youth's ability to recognize and express their emotion.

Exploring family psychiatric history was a primary aim of this study, as it is not been extensively studied previously in PNES youth. In this study, there were a significant number of first degree relatives, parents and siblings, who endorsed historically experiencing a psychiatric disorder. Anxiety and depressive disorders were the most commonly reported in this sample. Interestingly, seven families also had one or more first degree relative with history of conversion disorder. There are no known studies at the time of this publication that examine history of conversion disorder in families of PNES youth. Looking to the literature on chronic pain: parental psychopathology, how parents manage past trauma, and parent's somatic complaints (a form of dissociation), are associated with increased chronic pain in their children (9, 21, 27). Parental modeling of response to pain, reinforcement of illness behaviors in themselves and their children, and higher somatization rates are related to somatic symptoms in their children with chronic pain (24, 28). Parent's response to pain and experience of chronic pain conditions has also been associated with greater child functional impairment (18, 24, 27, 28). Though chronic pain disorders are not synonymous with PNES conversion symptoms, there is known overlap in the psychosomatic nature of some pain and conversion disorders (24). Looking to this literature to further the research and examination of this history in PNES may help to answer the questions about genetic predisposition for this disorder, the role of modeling in the disorder, or both.

This descriptive study is limited in that the examination of family psychiatric history was broad, looking only at general categories (anxiety, depression, etc.) without further classifying diagnosis, length or severity. While individual youth risk factors were assessed, family stressors outside of medical and psychiatric diagnoses were not recorded. Another significant limitation was the absence of a database with age-matched epilepsy controls who were evaluated at the time of diagnosis with the same comprehensive model applied to the PNES patients (namely evaluation by a clinical psychologist). This future study could allow for a much broader evaluation of both the youth's risk factors as well as family factors that contribute to the disorder.

In conclusion, the influence of these personal and family risk factors in the youth with PNES is an important area for future research. While individual risks have been documented in numerous studies (5–8), family risks and their influence on the youth, have not been well evaluated. Future studies should pursue a more comprehensive evaluation of these factors to better understand the development and management of this complex disorder.

## Data availability statement

The raw data supporting the conclusions of this article will be made available by the authors, without undue reservation.

## Ethics statement

The studies involving human participants were reviewed and approved by Children's Hospitals and Clinics of Minnesota Institutional Review Board. Written informed consent from the participants' legal guardian/next of kin was not required to participate in this study in accordance with the national legislation and the institutional requirements.

## Author contributions

Patient recruitment, evaluation, management of data, analysis of data, and generation of this manuscript were all completed by JD.
